# Cardiovascular health: a cross-national comparison between the Maine Syracuse Study (Central New York, USA) and ORISCAV-LUX (Luxembourg)

**DOI:** 10.1186/1471-2458-14-253

**Published:** 2014-03-15

**Authors:** Georgina E Crichton, Merrill F Elias, Adam Davey, Nicolas Sauvageot, Charles Delagardelle, Jean Beissel, Ala’a Alkerwi

**Affiliations:** 1 Nutritional Physiology Research Centre, University of South Australia, Adelaide, Australia; 2 Centre de Recherche Public Santé, Centre d’Etudes en Santé, Strassen, Grand-Duchy of Luxembourg; 3 Department of Psychology, University of Maine, Orono, ME, USA; 4 Graduate School of Biomedical Sciences and Engineering, University of Maine, Orono, ME, USA; 5 Department of Public Health, Temple University, Philadelphia, PA, USA; 6 Service de Cardiologie, Centre Hospitalier du Luxembourg, Luxembourg, Grand-Duchy of Luxembourg

**Keywords:** Cardiovascular disease, Cross-national comparison, Ideal cardiovascular health, Luxembourg, USA

## Abstract

**Background:**

Cardiovascular disease is the number one cause of death in the United States and in most European countries. Cardiovascular health, as defined by the American Heart Association, is comprised of seven health metrics (smoking, body mass index, physical activity, diet, total cholesterol, blood pressure, and fasting plasma glucose). No studies have compared US data with data collected elsewhere, using this index of cardiovascular health

**Methods:**

We performed comparative analyses of cardiovascular health status in participants from 2 study sites in 2 different countries: the Maine-Syracuse Study, conducted in Central New York, USA in 2001–2006 (n = 673), and the Observation of Cardiovascular Risk Factors in Luxembourg, conducted in 2007–2009 (n = 1145).

**Results:**

The Cardiovascular Health Score, the sum of the total number of metrics at ideal levels, was higher in the Luxembourg site than in the Central New York site. Ideal cardiovascular health levels for body mass index, smoking, physical activity, and diet were more prevalent in the Luxembourg site than the Central New York site. Ideal levels for blood pressure were more prevalent in Central New York. Differences between the two sites remained with control for age, gender and socioeconomic indicators.

**Conclusions:**

Cardiovascular health, as indexed by seven health metrics, was higher in the European study site than in the US study site. The largest differences were for the four lifestyle/behavior metrics, namely body mass index, smoking, physical activity, and diet. Preventative and intervention strategies will continue to be important for both countries in order to improve cardiovascular health.

## Background

Cardiovascular disease (CVD) accounts for approximately one of every three deaths in the United States, and is estimated to cost $312 billion annually [1]. The American Heart Association (AHA), in its recently released ‘Strategic Impact Goal Through 2020 and Beyond’ defined levels of four health behaviors (not smoking, engaging in sufficient physical activity, consuming a healthy diet, and body mass index (BMI) less than 25 kg/m^2^), and three health factors (optimal total cholesterol, blood pressure (BP), and fasting blood glucose), to identify ideal cardiovascular health [2]. A number of investigators have used this construct and reported low prevalences of ideal cardiovascular health in US samples [3-5]. Furthermore, negative correlations between ideal cardiovascular health and all-cause and CVD mortality [4,6], and cardiovascular events [4,5,7,8] have been identified. Our literature search showed little evidence that attention has been paid to this concept outside of the USA. Moreover, comparisons between the prevalence of poor, intermediate and ideal cardiovascular health levels at study sites in the USA and other countries have not been undertaken.

In order to make a cross-national comparison, we used data collected from two prominent studies with similar data on CVD risk factors, health behaviors, and demographic variables: the Maine-Syracuse Longitudinal Study (MSLS) in the United States, and the Observation of Cardiovascular Risk Factors in Luxembourg (ORISCAV-LUX). The MSLS was conducted in Syracuse, New York (NY), USA and its catchment area (Central NY). ORISCAV-LUX was a nation-wide, population-based study. Several considerations make these specific comparisons important and meaningful. First, existing multinational data collection efforts (e.g., Health and Retirement Study in the USA/Survey of Health, Ageing and Retirement in Europe) do not measure key aspects of cardiovascular health (e.g., diet, total cholesterol, BP) and we know of no nationally representative data set that permit comparisons on these indices. Second, the MSLS and ORISCAV-LUX collect measures in essentially identical fashions, and both samples are representative of their respective geographic areas. Data collected from Luxembourg are of particular interest due to the multinational nature of its population, with a large number of individuals originating from neighboring European countries living in Luxembourg. As in the US, CVD is the number one cause of mortality in Luxembourg, with CVD related illnesses accounting for 33.8% of all deaths in 2011 [9].

Our overall objective was to compare the pattern of cardiovascular health between two geographically and culturally distinct sites in the US and in Luxembourg. Specifically, the first objective was to compare the sites with respect to the total number of health metrics at ideal levels, indexed by a global Cardiovascular Health Score (CHS), derived from the AHA construct of cardiovascular health. The second objective was to examine the prevalence of poor, intermediate, and ideal health for each health behavior (smoking, physical activity, diet, BMI), and health factor (total cholesterol, BP, and fasting blood glucose) at each site. The third objective was to assess whether any observed differences in the cardiovascular health components between the two study sites remained after controlling for socioeconomic indicators. As European countries have higher rates of walking, cycling, and active transportation than in the United States [10], and a number of studies have shown an inverse association between active transportation and overweight/obesity, BP, and triglyceride and fasting insulin levels [10-15], we hypothesised that the CHS would be higher in the Luxembourg site than in the Central NY site. With regard to the individual health metrics, we postulated that the prevalence of ideal levels for physical activity and BMI would also be higher in Luxembourg than Central NY.

## Methods

### Participants in MSLS (USA)

The MSLS is a community-based study of aging, cardiovascular risk factors and cognitive functioning in adults, aged 23–98 years [16-19]. At initial recruitment, the sole exclusions were institutionalized people, diagnosed alcoholism and psychiatric disorder. The data for the present study were taken from subjects returning for the 6th (2001–2006) study wave when dietary intake measures were first obtained. At this time, 80.7% of participants were residing in Onondaga County and 10.1% in 3 counties surrounding Onondaga County in the Central NY area of upstate New York State. The remaining original residents of Central NY (9.2%) had relocated to 1 of 15 different states at the time this study was conducted. Demographics and health statistics published for Onondaga County, NY, best describe this sample [20]. Beginning with a sample of 1049 individuals, participants were excluded from the present analysis for the following reasons: missing dietary or cardiovascular health data (n = 34), acute stroke (n = 28), probable dementia (n = 8), hemo-dialysis (n = 5), inability to read English (n = 1), and alcohol abuse after baseline (n = 1), leaving 972 participants.

### Participants in ORISCAV-LUX (Luxembourg)

ORISCAV-LUX, a nationwide, cross-sectional study conducted in 2007–2009, was designed to gather information on the prevalence of cardiovascular risk factors among the adult population of Luxembourg. Exclusions were institutionalized people (n = 12), pregnancy (n = 21), serious mental and/or physical handicap (n = 5), prisoners (n = 1), people outside the determined age range (n = 2) and those deceased before recruitment (n = 5) [21]. A representative random sample of 1432 individuals, stratified by sex, age (18–69 years) and district of residence completed the recruitment procedure [21,22]. After eliminating those with missing data on components of cardiovascular health, data were available for 1352 of the ORISCAV-LUX sample.

### Final comparative sample

In both studies only participants aged 30–69 were included in order to compare two age-homogeneous samples. The final sample comprised 1818 individuals (673 from MSLS and 1145 from ORISCAV-LUX). Further details related to the methods of sampling for both studies appear elsewhere [18,19,21,22]. All participants gave informed written consent to take part. The MSLS was approved by the University of Maine Institutional Review Board and ORISCAV-LUX by the National Research Ethics Committee and the National Commission for Private Data Protection.

### Procedure

#### Cardiovascular health metrics

Participants at both study sites underwent physical and anthropometric measurements, blood tests, and completed self-administered questionnaires to gain information on demographic and socioeconomic characteristics. Standardized protocols for data collection were used. Body weight, height, BMI and BP measures were assessed as described previously for both studies [16,18,19,21-23]. Standard assay methods were employed [18,23] to obtain fasting plasma glucose (mg/dl) and total cholesterol (mg/dl).

In the MSLS, physical activity was measured with the Nurses’ Health Study (NHS) Activity Questionnaire [24]. Dietary intake was assessed using the food frequency questionnaire (FFQ) component of the Nutrition and Health Questionnaire [25]. Smoking status was based on self-report from the same questionnaire [25]. At the conclusion of each wave, MSLS subjects were informed of any new risk factors detected at that examination and advised to consult their physician for treatment.

In ORISCAV-LUX, physical activity was measured using the short format International Physical Activity Questionnaire (IPAQ) [26]. Detailed data regarding smoking were obtained from the health questionnaire. Dietary intake was assessed using a semi-quantified FFQ assessing the frequency of consumption of 134 items [27]. All questionnaires used in ORISCAV-LUX were available in French, English, German, and Portuguese. ORISCAV-LUX subjects were advised to see their physicians for treatment if any cardiovascular anomaly was detected during the study assessment.

#### Diet metric

For the diet metric, two food scores were calculated for each study, a Recommended Food Score (RFS) [28], and a non-Recommended Food Score (non-RFS) [29]. These scores were used according to the availability of dietary data in order to capture a detailed measure of dietary intakes. The RFS comprised 18 food items, based on the recommendations of the 2010 Dietary Guidelines for Americans [30]. One point was awarded for consumption of any of the recommended foods at least once per week (fruit, vegetables, legumes, wholegrain cereal products, low fat dairy products, fish, nuts), otherwise 0 points were given [28], to give a total score out of 18. Included foods were similar to those used previously [28,31,32].

The non-RFS [29] included 13 items that are recommended to reduce [30], including processed meats, refined grains, solid fats, added sugars, and alcohol. Consumption of non-recommended foods at least two to four times per week was assigned a score of 1; otherwise 0 points were assigned [31,33]. A total non-RFS out of 13 was calculated, with a higher value indicating a higher consumption of non-recommended food items.

#### Cardiovascular health score

Poor, intermediate, and ideal health levels for smoking, BMI, physical activity, total cholesterol, BP, and fasting plasma glucose were calculated using the AHA definitions [2] (see Online Resource 1). For the RFS, scores of 0–7, 8–11, and 12–18 were defined as poor, intermediate, and ideal, respectively. Scores of 5–13, 3–4, and 0–2 for the non-RFS were defined as poor, intermediate, and ideal.

The CHS comprised the sum of components at ideal levels, ranging from 0 (no cardiovascular health components at ideal levels) to 8 (all cardiovascular health components at ideal levels). This global score was then categorized into low (0–2 components at ideal levels), medium (3–5) or high (6–8).

### Data analysis

According to the type of variable (continuous or categorical), independent samples t-tests and Chi-square tests were used to compare demographic variables, mean scores of the cardiovascular health metrics and other health variables in the two samples (n = 673 for MSLS, n = 1145 for ORISCAV-LUX).

The proportion of each sample in the poor, intermediate and ideal categories for each cardiovascular health metric were calculated. As prevalence estimates are strongly gender and age-dependent (e.g., risks increase for chronic diseases with age), prevalence comparisons between populations may be misleading if the underlying age and/or gender composition differs in the populations being compared [34]. To overcome this, direct gender and age standardization according to the Segi world standard population was used to compute gender and age standardized prevalence in each health category [35].

Prevalence of each health category (e.g., poor health) for each health metric in the two study sites were compared by computing the comparative morbidity ratio and testing it for statistical significance [36]. This was also performed for the global CHS (comparing low, medium and high categories between the two sites).

General linear models were used to examine associations between study site and each cardiovascular health component (as continuous variables). Statistical adjustment was made for age, gender, education and income. Interactions of study site with gender and age were not significant. SPSS version 21 and SAS (version 6.1) were used for all analyses. P values of <0.05 were considered statistically significant.

Sensitivity analyses were performed excluding the minority populations in each sample (n = 52 in ORISCAV-LUX and n = 65 in MSLS) and excluding the persons residing in states other than NY at the time of wave 6 MSLS (10%). The results were unchanged (data not shown).

## Results

### Descriptive data

Table 1 shows the demographic, cardiovascular health components, and other health variables for MSLS and ORISCAV-LUX participants. The MSLS sample had a higher proportion of participants who were obese, had diagnosed hypertension, diabetes, or CVD (all p < 0.001), compared with the Luxembourg sample. The proportion of those treated for diagnosed hypertension was significantly higher at the Central NY site (84.3%) than in Luxembourg (37.7%) (p < 0.001).

**Table 1:** Demographic, cardiovascular and health variables for MSLS (n = 673) and ORISCAV-LUX (n = 1145) participants

**Variable**	**Central New York MSLS**		**Luxembourg ORISCAV-LUX**		**p-value**
	**M or %**	**SD**	**M or %**	**SD**	
Age, yrs	55.5	8.7	47.9	10.7	<0.001
Gender					0.001
Males	41.0		48.8		
Females	59.0		51.2		
Education					<0.001
Primary	5.4		26.8		
Secondary	40.1		46.7		
Tertiary	54.5		26.5		
Income^1^					<0.001
Q1 (< 30 000)	23.8		17.8		
Q2 (30 000 to 60 000)	37.6		51.0		
Q3 (60 000–120 000)	35.3		25.8		
Q4 (>120 000)	3.3		5.4		
Race					<0.001
Caucasian	90.3		95.5		
Other	9.7		4.5		
Cardiovascular Health metrics					
Smoking, no cigarettes per day (all)	1.7	5.7	2.7	7.2	0.002
Smoking, no cigarettes per day (smokers)	13.0	10.2	14.6	10.3	0.23
BMI, kg/m^2^	29.9	6.3	27.1	4.9	<0.001
Physical activity, mins/wk^2^	273	345	778	936	<0.001
Total cholesterol, mg/dl	203.6	39.8	206.3	39.7	0.17
Systolic BP, mm Hg	126.8	20.7	131.6	18.2	<0.001
Diastolic BP, mm Hg	70.8	10.2	83.8	10.9	<0.001
Fasting blood glucose, mg/dl	98.4	28.9	94.5	18.6	<0.001
RFS, 0-18^3^	9.2	2.8	10.8	2.8	<0.001
non-RFS, 0-13^4^	3.3	1.6	3.0	1.6	<0.001
Total CHS, 0-8	3.8	1.6	4.2	1.6	<0.001
Proportion of sample with CHS 0/8	0.7		0.3		0.25
Proportion of sample with CHS 8/8	0.4		1.0		0.17
Other health measures					
Height, cm	168.7	9.8	169.4	9.8	0.13
Weight, kg	85.3	20.2	78.1	16.5	<0.001
Waist circumference, cm	95.6	15.6	91.4	13.5	<0.001
Hip circumference, cm	109.2	12.5	101.6	9.5	<0.001
Waist:hip	0.87	0.09	0.90	0.09	<0.001
CRP, mg/dL	0.41	0.46	0.27	0.48	<0.001
Alcohol intake, standard drinks/d	0.5	1.0	1.3	1.3	<0.001
Obese, BMI ≥ 30 kg/m^2^	42.1		25.5		<0.001
Diabetes^5^	10.7		5.8		<0.001
Treated participants with diabetes	79.2		65.2		0.07
Treated diabetes: fasting blood glucose, mg/dl	156.3	65.3	152.7	45.4	0.76
Untreated diabetes: fasting blood glucose, mg/dl	143.7	21.3	138.4	15.1	0.37
Hypertension^6^	54.8		43.3		<0.001
Treated participants with hypertension	84.3		37.7		<0.001
Treated hypertension: systolic BP, mm Hg	134.4	20.1	145.4	18.7	<0.001
Treated hypertension: diastolic BP, mm Hg	73.4	9.4	89.2	11.3	<0.001
Untreated hypertension: systolic BP, mm Hg	152.3	13.1	146.0	14.9	0.003
Untreated hypertension: diastolic BP, mm Hg	81.2	11.1	93.5	8.5	<0.001
CVD^7^	11.3		4.0		<0.001

^1^US $ or euros per year.

^2^Includes moderate and vigorous physical activity.

^3^Higher scores indicate higher intake of recommended foods.

^4^Higher scores indicate higher intake of non-recommended foods.

^5^Fasting plasma glucose ≥ 126 mg/dL or taking anti-diabetic medication.

^6^Had systolic BP ≥ 140 mmHg and/or diastolic BP ≥ 90 mmHg or taking anti-hypertensive medication.

^7^Includes myocardial infarction, coronary artery disease, heart failure, angina pectoris, transient ischemic attack.

### Cardiovascular health

#### Health behaviors

As shown in Table 1, BMI was significantly higher in the Central NY sample than in the Luxembourg sample (p < 0.001). The dietary measures indicated a lower intake of recommended foods and a higher intake of non-recommended foods at the Central NY site compared with intakes at the Luxembourg site (both p < 0.001). Cigarettes smoked per day and time spent engaging in weekly physical activity were both higher at the Luxembourg site than at the Central NY site (both p < 0.01).

The observed differences in BMI, physical activity and diet (RFS and non-RFS) remained statistically significant with the additional adjustment for age, gender, education, and income (all p < 0.001) (Table 2). Weekly physical activity time was over two times higher in the Luxembourg sample than in the Central NY sample. This difference remained significant in secondary analyses with statistical adjustment for BMI, waist circumference and waist/hip ratio (data not shown).

**Table 2:** **Multivariate-adjusted means**^**1 **^**and SE for total Cardiovascular Health Score and cardiovascular health metrics, MSLS (n = 673) and ORISCAV-LUX (n = 1145)**

**Cardiovascular Health variable**	**Central New York MSLS**		**Luxembourg ORISCAV-LUX**		**Mean difference**	**p-value**
	**M**	**SE**	**M**	**SE**		
Total Cardiovascular Health Score, 0-8	3.8	0.08	4.2	0.05	−0.38	<0.001
Cardiovascular Health metrics						
Smoking, no cigarettes per day	2.3	0.34	2.3	0.21	−0.04	0.93
BMI, kg/m^2^	29.9	0.26	27.0	0.17	2.89	<0.001
Physical activity, mins/wk^2^	358	46	754	26	−396	<0.001
Total cholesterol, mg/dl	201.3	2.04	207.4	1.30	−6.08	0.017
Systolic BP, mm Hg	122.7	0.85	132.7	0.54	−9.94	<0.001
Diastolic BP, mm Hg	70.1	0.52	83.9	0.33	−13.85	<0.001
Fasting blood glucose, mg/dl	96.1	1.03	94.7	0.65	1.37	0.29
RFS, 0-18^3^	8.9	0.14	10.9	0.09	−2.0	<0.001
non-RFS, 0-13^4^	3.5	0.08	3.0	0.05	0.55	<0.001

^1^Adjusted mean values for age, gender, education and income.

^2^Includes moderate and intense physical activity.

^3^Higher scores indicate higher intake of recommended foods.

^4^Higher scores indicate higher intake of non-recommended foods.

#### Health factors

Fasting plasma glucose was significantly higher in the Central NY sample than in the Luxembourg sample (p < 0.001), while mean systolic and diastolic BP were both significantly higher in Luxembourg (both p < 0.001) (Table 1). BP measures, as well as total cholesterol were significantly higher in Luxembourg than in the Central NY site when adjusted for age, gender, education, and income (Table 2). The BP difference was unchanged with statistical adjustment for BMI, waist circumference and waist/hip ratio (data not shown).

#### Total cardiovascular health score

The mean total CHS (number of metrics at ideal levels) was significantly higher at the Luxembourg site (4.2) than at the Central NY site (3.8) (fully adjusted model, Table 2, p < 0.001). The proportion of the sample with overall ‘ideal health’ (8/8 components at ideal levels) was 1.0% at the Luxembourg site, compared with 0.4% at the Central NY site (not statistically significant).

### Poor, intermediate and ideal cardiovascular health comparison

The percentages of participants in ORISCAV-LUX and MSLS with poor, intermediate and ideal health for each health behavior and factor, and the global CHS (age and gender standardized) are shown in Table 3. The percentage of those with ideal health was significantly higher in the Luxembourg site than in the Central NY site for BMI, smoking, physical activity and diet (both scores) (all p < 0.01). The percentage of those with ideal BP levels was higher in the Central NY site than in the Luxembourg site (p < 0.001).

**Table 3:** Age- and gender-standardized proportions of participants in the MSLS (n = 673) and ORISCAV-LUX (n = 1145), with poor, intermediate and ideal health for each health component, and for the global Cardiovascular Health Score

**Cardiovascular Health component**	**Definition**^**1**^	**Central New York MSLS (%)**	**Luxembourg ORISCAV-LUX (%)**	**p-value**
BMI				
Poor	≥30 kg/m^2^	43.0	25.4	<0.001
Intermediate	25-29.9 kg/m^2^	34.5	35.0	0.88
Ideal	<25 kg/m^2^	22.5	39.6	<0.001
Smoking				
Poor	Current smoker	16.6	18.8	0.43
Intermediate	Former smoker, quit < 12 months	54.5	28.6	<0.001
Ideal	Never or quit > 12 months	29.0	52.6	<0.001
Physical activity				
Poor	No physical activity	10.6	11.9	0.58
Intermediate	1-149 min/wk moderate intensity activity^2^	30.0	19.8	0.001
Ideal	≥ 150 min/wk moderate intensity activity^3^	59.5	68.3	0.013
Fasting plasma glucose				
Poor	≥126 mg/dL	5.6	4.8	0.48
Intermediate	100-125 mg/dL or treated to goal	15.4	21.4	0.021
Ideal	<100 mg/dL	79.0	73.8	0.056
Total cholesterol				
Poor	≥240 mg/dL	11.6	19.6	<0.001
Intermediate	200-239 mg/dL or treated to goal	37.4	33.5	0.20
Ideal	<200 mg/dL	51.0	46.9	0.21
Blood pressure				
Poor	Systolic BP ≥140 or diastolic BP ≥90 mm Hg	18.4	38.5	<0.001
Intermediate	Systolic BP 120–139 or diastolic BP 80–80 mm Hg or treated to goal	33.0	38.6	0.10
Ideal	<120/80 mm Hg	48.6	23.0	<0.001
RFS^4^				
Poor	0-7	34.5	11.8	<0.001
Intermediate	8-11	48.8	45.5	0.34
Ideal	12-18	16.7	42.8	<0.001
non-RFS^5^				
Poor	5-13	24.9	18.4	0.047
Intermediate	3-4	45.5	42.7	0.41
Ideal	0-2	29.6	38.9	0.005
Global CHS				
Low	0-2 components in ideal range	19.6	15.2	0.12
Medium	3-5 components in ideal range	65.2	64.1	0.76
High	6-8 components in ideal range	15.2	20.6	0.049

^1^Definitions according to the American Heart Association except for diet score, for adults >20 years of age.

^2^Or 1–74 min/wk vigorous intensity activity.

^3^Or ≥75 min/wk vigorous intensity activity.

^4^RFS scored out of 18, with higher scores indicating a higher consumption of recommended foods to increase.

^5^non-RFS scored out of 13, with higher scores indicating a higher consumption of foods recommended to reduce.

Poor health levels for BMI and diet (RFS and non-RFS) were statistically significantly higher at the Central NY site than at the Luxembourg site (all p < 0.05). Prevalence of poor levels for BP and total cholesterol were higher in Luxembourg than in Central NY (both p < 0.001).

**Figure 1:**
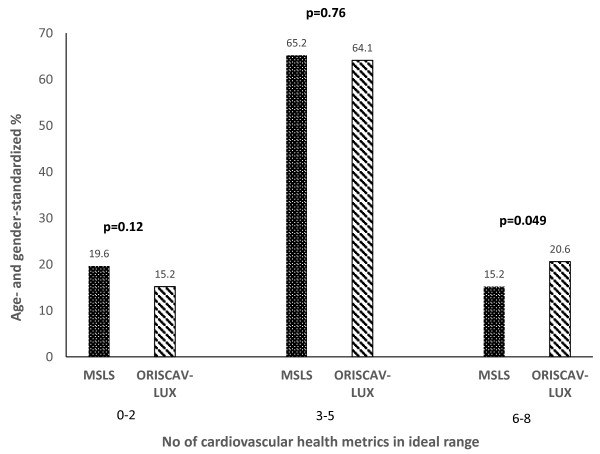
**Proportion of Maine-Syracuse Longitudinal Study (MSLS) and Observation of Cardiovascular Risk Factors in Luxembourg (ORISCAV-LUX) participants (aged 30–69 years) with low (0–2 health components at ideal levels), medium (3–5 health components at ideal levels), and high (6–8 health components at ideal levels) Cardiovascular Health Score.** p-values represent significant differences in proportions between the 2 sites.

Figure 1 shows the proportion of participants in each site (aged 30–69 years) with a low (0–2 health components at ideal levels), medium (3–5 health components at ideal levels), and high (6–8 health components at ideal levels) CHS. A greater percentage of participants in the Luxembourg site had a higher number of total health components in the high category (CHS 6–8), than in the Central NY site (p < 0.05).

## Discussion

The present study is, to our knowledge, the first to make cross-national comparisons using the AHA-defined components of cardiovascular health. Although we are unable to generalize beyond the two geographic study sites in Central NY, USA and Luxembourg, the study provides insight into how cardiovascular health differs between two sites in the US and Europe. Luxembourg, a centrally located European country with a large proportion of the population coming from Portugal and neighboring countries including Belgium, Germany and France, serves as a good representation of western Europe. Syracuse and its surrounding counties are ethnically diverse, and constitute the economic and educational hub of Central NY state.

The overall CHS, generated from the individual health metrics, was higher at the Luxembourg site than at the Central NY site. Ideal levels for BMI, smoking, physical activity, and diet were more prevalent in Luxembourg than in Central NY. However, ideal levels for BP were more prevalent in Central NY. Differences between the two sites with respect to BMI, physical activity, diet and BP cannot be attributed to age, gender, income and education as findings remained after adjustment for these potential confounders.

Importantly, the prevalence of overall ideal cardiovascular health (ideal levels for all components) was low at both the Central NY site (0.4%) and the Luxembourg site (1.0%). This is consistent with smaller state-based studies and national data in the USA, with prevalences ranging from 0–0.1% [1,3,5] to 1.2% [4]. Of concern are the national (US) data indicating that the prevalence of ideal cardiovascular health decreased from 2.0% in 1988–1994 to 1.2% in 2005–2010 [4]. Over the same time, increases in physical inactivity and obesity, and decreases in fruit and vegetable consumption have been observed [37]. The biggest prevalence differences between the two present studies were observed for the RFS. Poor health for this score in the Central NY sample (34.5% of participants) was nearly three times higher than in Luxembourg (11.8% of participants).

The role of physical activity in weight control is well established. While the age-adjusted proportion of participants not engaging in any physical activity was similar in both study sites (10-12%), the mean time spent engaging in physical activity per week was over two times higher in the Luxembourg site than Central NY, equating to a difference of approximately 6.5 hours per week. Adjusting for BMI, waist circumference and waist/hip ratio in secondary analyses did not change the results (data not shown). This is consistent with data showing that Europeans walk and cycle over two and four times, respectively, the number of kilometres per person per year, than residents of the United States [10]. Furthermore, active transportation via walking or cycling is also more common in Europe than in North America and the lowest estimates of adult obesity are found in countries that rely more upon active transportation and less upon automobiles [10,11]. As per intuition, higher rates of walking and cycling as a means of transport have also been associated with a higher percentage of adults meeting the recommended levels of physical activity, as well as lower estimates of diabetes [11]. The infrastructure in Luxembourg supports walking and cycling for daily travel and may be one contributing factor that explains the difference observed in the present study. During the period of data collection for MSLS, the automobile was the predominant mode of transportation in Syracuse and Central New York with few cycling paths on city and town streets.

In contrast to these findings, average age and gender-adjusted BP was 133/84 mmHg in the Luxembourg sample compared with 123/70 mmHg in the Central NY sample. Epidemiological research utilizing national survey data from six European countries, the US and Canada, also found higher BP levels in Europe, and reported very similar levels to those in the present study: 136/83 mmHg in Europe and 127/77 mmHg in North America [38]. Of note, less than 40% of those with diagnosed hypertension in the Luxembourg sample were being treated for high BP, compared with nearly 85% in the MSLS. Although hypertension has the same classification in the US and Europe (systolic BP ≥140 mmHg and/or diastolic BP ≥90 mmHg) [39,40], the initial approaches to BP control vary and we can only speculate that this may contribute to the BP differences observed [39,40]. Lifestyle modification and immediate initiation of antihypertensive drug therapy is recommended in the US [40], whereas in Europe, the immediate initiation of drugs needs the presence of other symptomatic CVD risk factors [39].

In addition, poor awareness of relatively ‘silent’, asymptomatic cardiovascular risk factors including hypertension and dyslipidemia has been demonstrated in this Luxembourg population, with 60% unaware of their diagnosed hypertension (diagnosis made from the ORISCAV-LUX survey) [41]. This level of unawareness is two-fold that of the US; national estimates from the National Health and Nutrition Examination Survey 1999–2000 data indicate an unawareness level of 30% [40].

There are several study limitations. ORISCAV-LUX was a community, national population-based study, whereas MSLS was a community-based sample restricted to Central NY. The MSLS sample is not nationally representative, but both Luxembourg and the Syracuse metropolitan statistical area (MSA) have similar population sizes of slightly more than half a million inhabitants. In 2008, Central NY state had an almost identical age-adjusted CVD mortality estimate as the US (244.0 versus 244.8 per 100,000 deaths [42,43]. In Onondanga County, NY, CVD mortality estimates have been decreasing, following a national trend [42,43]. While African Americans have been found to have significantly fewer ideal cardiovascular health components than whites [3,5], a sensitivity analysis excluding African Americans (9.7% in MSLS) did not affect the pattern of results. Smoking, diet and physical activity data were based on participant self-report and the same instruments were not used in both studies.

There are several study strengths. This is the first study to compare three levels of cardiovascular health in two studies from two different countries. We are not aware of published data using ideal cardiovascular health in countries outside of the USA. To compare prevalence estimates across these two samples with different age compositions, the effects of variation in age structure were removed by using a ‘world’ standard population to standardize age [34]. The prevalence differences observed in these analyses were confirmed when comparisons between the two sites were made using the health metrics as continuous variables, with the added control of education and income.

The purpose of our study was solely to describe the epidemiological patterns of cardiovascular health in these two study sites. The contrasts found are of substantial magnitude, however the findings neither provide causal explanations nor effectiveness data on the health care system in either region.

## Conclusions

The main finding of note in the present study is in the recognition that the majority of participants at both study sites had overall cardiovascular health scores that fell within the intermediate range, but the overall CHS was higher in Luxembourg. The differences found for the BP, diet, and physical activity metrics are particularly notable. As both Luxembourg and the New York State Department of Health have implemented public health policies to promote and maintain population cardiovascular health [44,45], it is difficult to attribute findings to specific health care policies, particularly as health care delivery is different in each region.

However the current findings suggest that different strategies for intervention will be important for different countries and underscore the need for cross-national comparisons. The continued improvement of education programs and focus on prevention measures may be helpful in both countries. Infrastructure to support active means of transportation may be an important consideration. Regardless of the approaches taken to achieve CVD reduction, even small health behavior changes at a population level would produce relatively large increases in the proportion of individuals in both ideal and intermediate categories [1]. In a similar vain, small reductions in weight gain over decades may accumulate into meaningful reductions in risk for obesity-related disorders [46]. The impact on US mortality from poor dietary habits is significant [47], yet simple changes, such as dietary reduction of 3 g of salt per day, is projected to yield substantial reductions in mortality and health care costs [48]. For example, the total costs of diagnosed diabetes in the US was estimated at $245 billion in 2012 [49], and costs approximately €148 million per year in Luxembourg [50]. Early intervention strategies to increasing physical activity, make healthier food choices, cease smoking, and lower blood sugar levels all seem important based on our findings, and may help to produce significant savings in health care costs in the long term. It may be particularly important for future studies to focus on children and young adults [51] in relation to interventions designed to raise the AHA cardiovascular health metrics to higher levels.

## Abbreviations

AHA: American Heart Association; BMI: Body mass index; CHS: Cardiovascular Health Score; CRP: C-reactive protein; CVD: Cardiovascular disease; FFQ: Food frequency questionnaire; IPAQ: International Physical Activity Questionnaire; MSLS: Maine-Syracuse Longitudinal Study; non-RFS: non-Recommended Food Score; NY: New York; ORISCAV-LUX: Observation of Cardiovascular Risk Factors in Luxembourg; RFS: Recommended Food Score.

## Competing interests

The authors declare that they have no competing interests.

## Authors’ contributions

GC: conceptualization of the present study, data analyses and interpretation, manuscript drafting. ME: MSLS chief investigator, manuscript drafting and revision. NS: statistical analyses and interpretation, critical review of manuscript. AD: critical review and drafting of manuscript. JB, CD: ORISCAV-LUX investigators, critical review of manuscript. AA: ORISCAV-LUX investigator, study design, data analyses and interpretation, critical review of manuscript. All authors read and approved the final manuscript.

## Pre-publication history

The pre-publication history for this paper can be accessed here:

http://www.biomedcentral.com/1471-2458/14/253/prepub
